# Supplementary material for: Identification and Validation of the lncRNA MYOSLID as a Regulating Factor of Necroptosis and Immune Cell Infiltration in Colorectal Cancer following Necroptosis-Related LncRNA Model Establishment
Source: Cancers (Basel). 2022 Sep 7;14(18):4364. doi: 10.3390/cancers14184364 (PMC9496742; doi:10.3390/cancers14184364)
Supplement: Supplementary file 1 [file cancers-14-04364-s001.zip › cancers-1822528-supplementary.pdf]

cancers

MDPI

Supplementary File

Identification and Validation of the lncRNA MYOSLID as a Regulating Factor of Necroptosis and Immune Cell Infiltration in Colorectal Cancer Following Necroptosis-Related LncRNA Model Establishment

Zhiwei Wu, Fan Zhang, Yaohui Wang, Zhixing Lu and Changwei Lin

Supplementary Information about the methods of In Vitro Expriments

Methods

CCK-8 and colony formation assay

The CCK8 (Cell Counting Kit 8) assay (Dojindo, Kumamoto, Japan) was used to measure cell proliferation in 96-well plates. RKO Cells were seeded at 5×10^3 cells per well, with five replicates for each condition. CCK8 was added at 0, 24, 48, 72, 96h and 120h and incubated at 37 °C for 2h. Cell numbers were determined by measuring the absorbance at 450 nm using a 96-well format plate reader . For colony formation assays, 500 cells were seeded into each well of a 6-well plate in triplicate for each condition and incubated for 14 days. Colonies were fixed with paraformaldehyde, stained with crystal violet and the colony numbers were counted. Average colony counts were calculated, and a paired t-test was used to test statistical significance. Each experiment was repeated three times.

5-. ethynyl-20-deoxyuridine (EdU) poliferation assays

For 5-ethynyl-20-deoxyuridine (EdU) staining, 5000 RKO cells were seeded and cultured in 96-well plates for 48 h, EdU reagent(cat. no. KGA337; KeyGen Biotech, China) was added at a final concentration of 10 μM and the plates incubated for 2 h at 37 °C. Then, cells were harvested. Then EdU and hochest 33258 staining according to the manufacturer's instructions.

Calcein-AM/PI viable and dead cells detection

The viable and dead cells were detected using a Calcein-Methyl 4-acetoxybenzoate (Calcein-AM)/Prodium Iodide (PI) kit (YEASEN, China). Briefly, the RKO cells were collected, and the supernatant was discarded. After being rinsed with assay buffer 3 times, the 100ul staining reagent was added to the cell mixture and incubated for 15 min at 37 °C。

Supplementary Information about the methods of In Vivo Expriments

Methods

Immunohistochemistry

To detect positive RIPK3, these tumor specimens were immobilized, embedded and created into 8-μm paraffin sections. The sections of these mice were deparaffinized in xylene and hydrated in a series of ethanol solutions. The sections were treated with citrate buffer (pH 6.0) at 96 °C for 20 min and incubated with an anti-RIPK3 antibody (Affinity, 1:200) overnight at 4 °C. The samples were washed and incubated with a corresponding secondary antibody (1:400) (Affinity) for 30 min at 37 °C. The sections were incubated with diaminobenzidine solution (Solarbio) and counterstained with hematoxylin (Solarbio).

Flow Cytometry Analysis of Tumor-Infiltrating Immune Cells

Tumors were excised from mice, weighted and mechanically diced in serum-free RPMI 1640 media. Tissue was digested in 200U/mL Collagenase Type IV (BioFroxx, 2091), 30 U/mL DNAse Type I (Biosharp, BS137), and 150U/mL Hyaluronidase Type V (MKBio, MX1007) at 37 °C for 30 min. Then, the cell suspension was filtered through 70-µm cell strainers twice and washed by PBS. To remove red blood cells, ACK lysis buffer (3× by volume) (Biosharp, BL503A) was added for 2 min followed by two volumes of RPMI to stop red cell lysis. After counting cells, these cells were blocked with TruStain FcX (anti-mouse CD16/32) antibody (BioLegend, 101319) at 4°C for 15 minutes and then incubated with Zombie Aqua Live/Dead fixable dye (BioLegend, 423102) at 4°C for 15 minutes. Then these cells were washed twice with PBS. Subsequently, specific antibodies recognized cell surface markers were stained with an antibody cocktail at 4°C for 30 minutes in a volume of 100 µl and washed twice with staining buffer (PBS supplemented with 1% BSA). Samples were acquired on a LSRII Fortessa (BD Biosciences) and analyzed using FlowJo software.

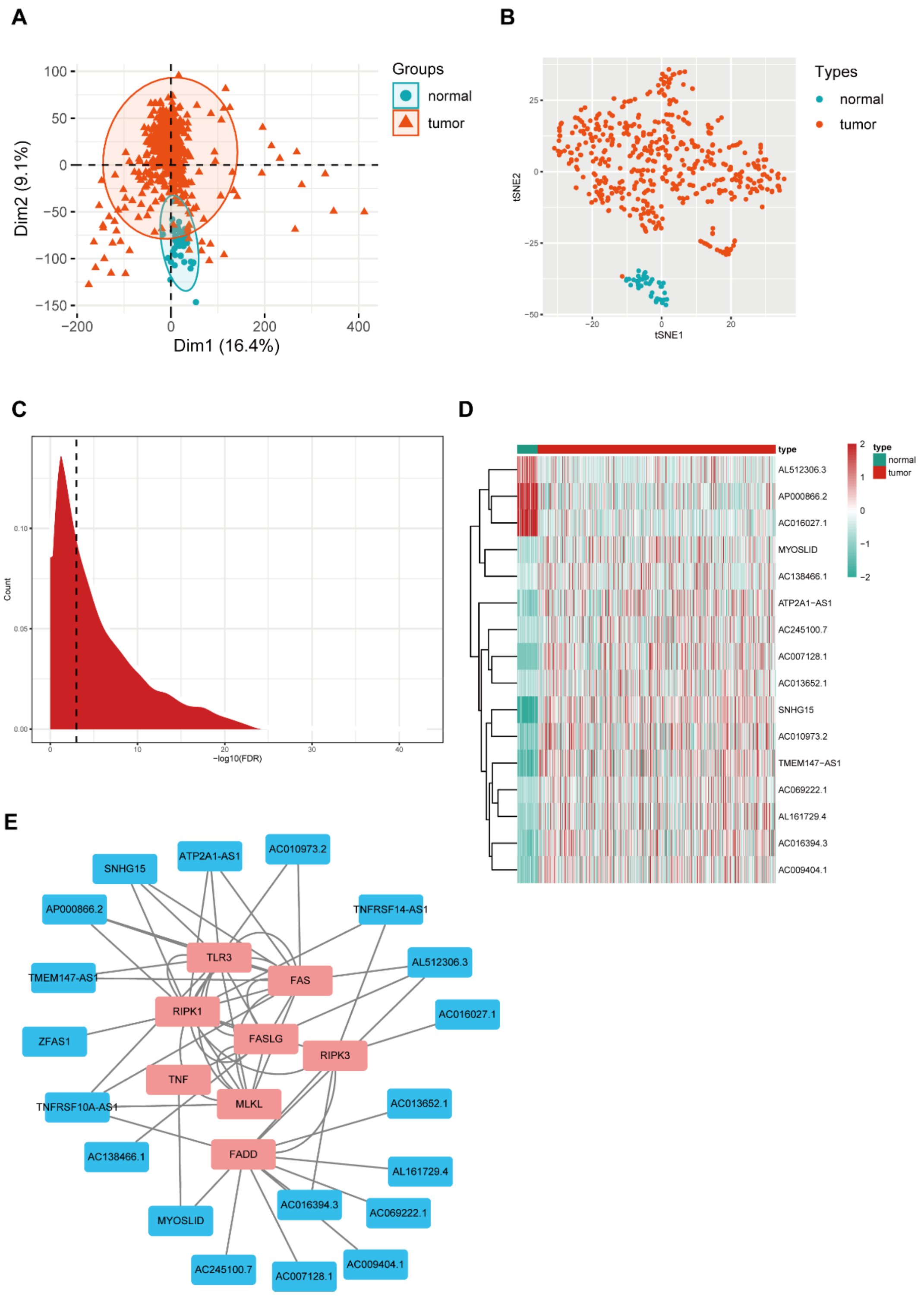

Figure S1. (A-C) A PCA map (A), a t-SNE (B) figure and a bar chart (C) showing the distribution of the TCGA samples. **(D)** A heatmap showing the expression level of 16 prognostic necroptosis-related differentially expressed lncRNAs in CRC samples from the TCGA database. **(E)** The coexpression network of 7 necroptosis-related genes and necroptosis-related differentially expressed lncRNAs.

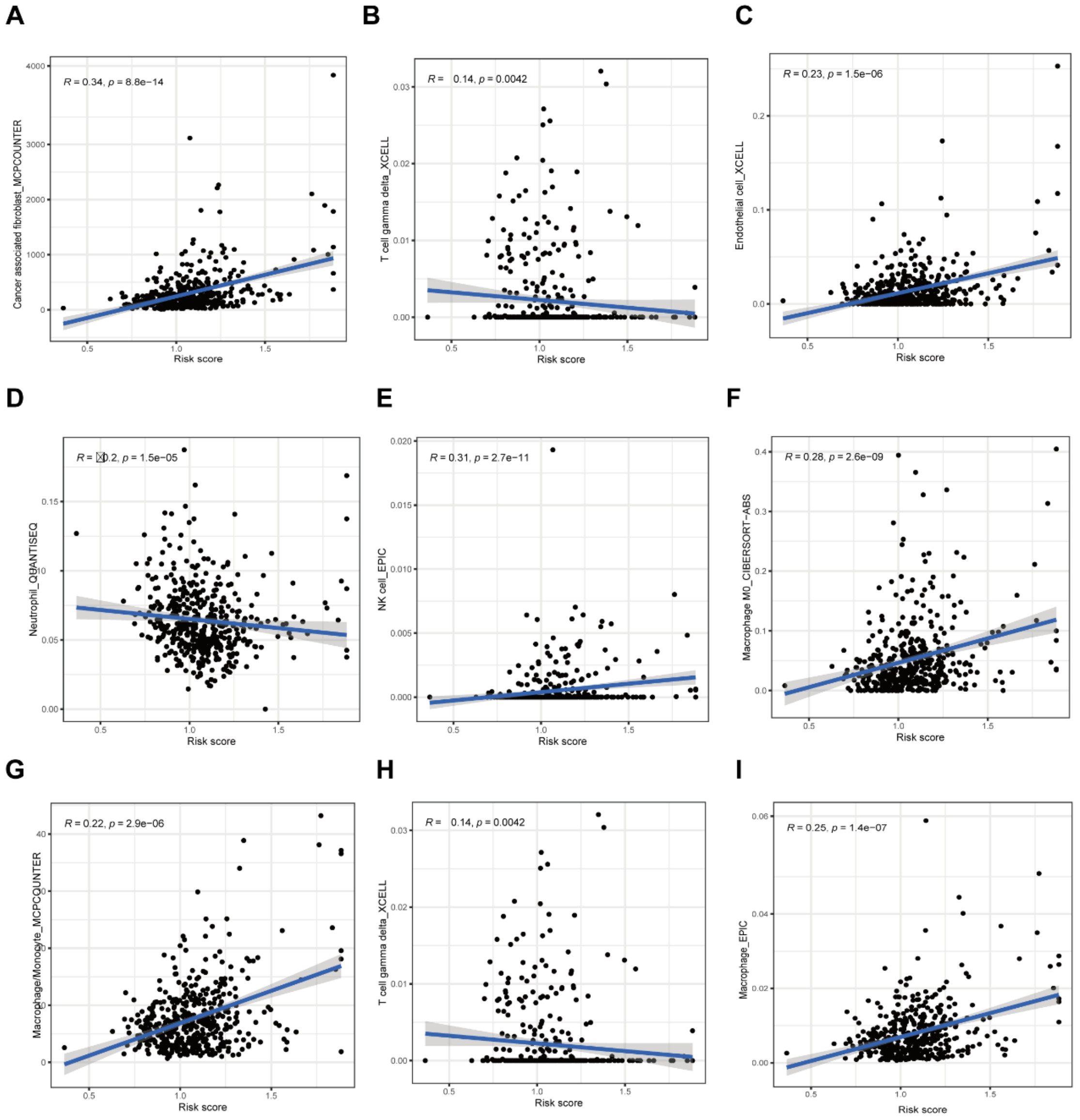

Figure S2. (A-I) The correlation between the risk score based on our model and immune cells, including cancer associated fibroblast (A), T cell gamma delta (B), endothelial (C), neutrophil (D), NK cells (E), Macrophage M0 (F), macrophage/monocyte (G), T gamma cell (H), and macrophage (I).

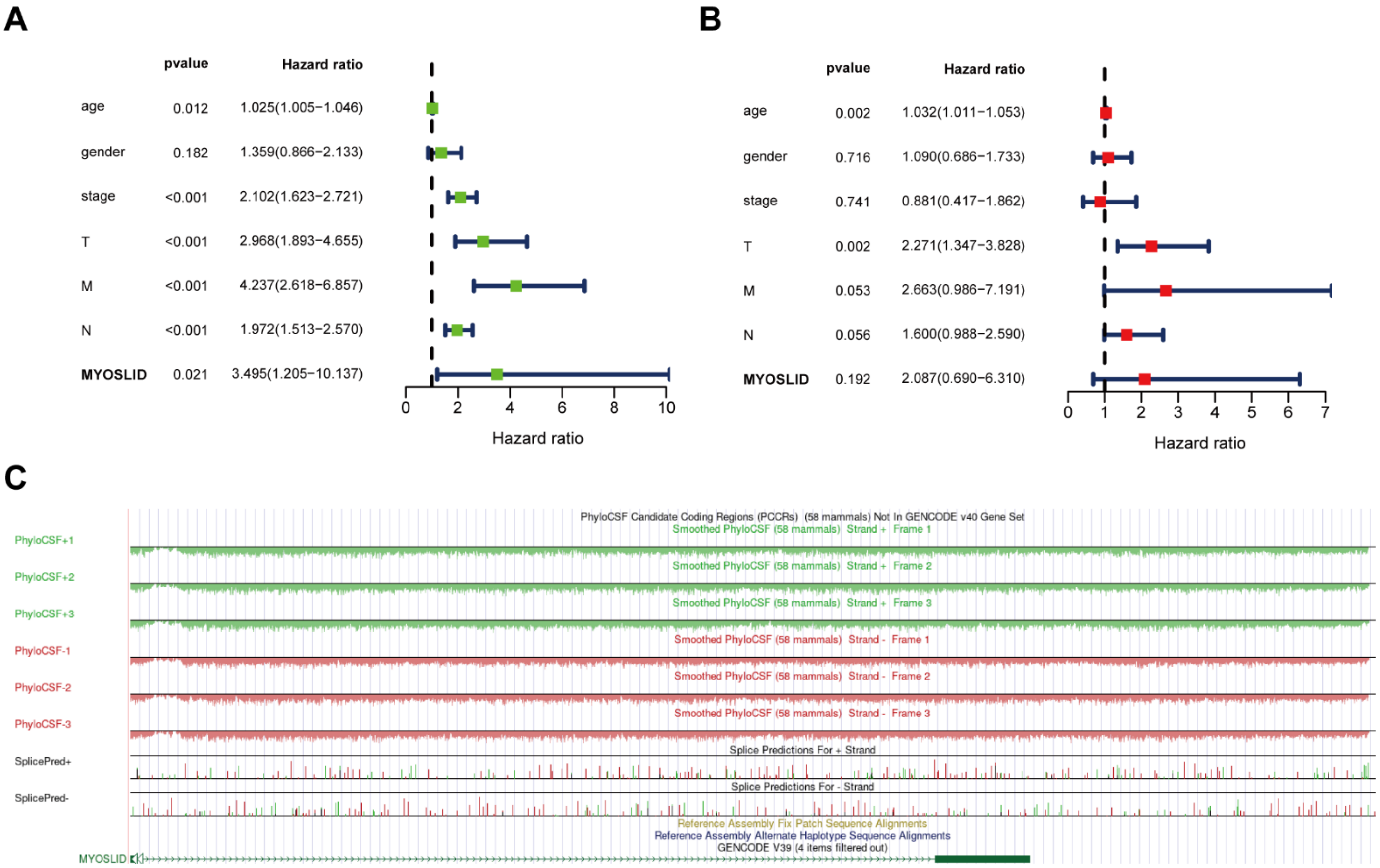

Figure S3. (A-B) Univariate and multivariate Cox analyses identified MYOSLID as a potential prognostic factor of CRC. (C) PhyloCSF determined that the coding potential of MYOSLID was limited.

Table S1. Primer sequences for MYOSLID.

Primers	Sequence
F-MYOSLID	AAGAGGGAGTGGGAGTTAGGC
R-MYOSLID	CACTGTGGTGGGATCTGCAAG
F-GAPDH	TGATGACCCTTTTGGCTCCC
R-GAPDH	GAAGCTTGTCATCAATGGAAAT

Table S2. The details of FADD, FAS, FASLG, MLKL, RIPK1, RIPK3, TLR3 and TNF.

Symbol	Ensembl	Description
FADD	ENSG00000168040	Fas Associated Via Death Domain
FAS	ENSG00000026103	Fas Cell Surface Death Receptor
FASLG	ENSG00000117560	Fas Ligand
MLKL	ENSG00000168404	Mixed Lineage Kinase Domain Like Pseudokinase
RIPK1	ENSG00000137275	Receptor Interacting Serine/Threonine Kinase 1
RIPK3	ENSG00000129465	Receptor Interacting Serine/Threonine Kinase 3
TLR3	ENSG00000164342	Toll Like Receptor 3
TNF	ENSG00000232810	Tumor Necrosis Factor

Table S3. The list of 421 necroptosis-related differentially expressed lncRNAs.

**Symbol**				
AL138820.1	AL512506.1	AC087683.2	AC011933.2	MIR22HG
AC010536.2	AC092828.1	AP002336.2	AC007128.1	AC016405.1
PKP4-AS1	AC074050.4	AC007216.3	AC024940.6	TBL1XR1-AS1
AC058791.1	AC008114.1	AC121761.1	AL136988.2	AC018410.1
ARAP1-AS2	AL512652.1	AC104785.1	FAM30A	AC137932.2
JARID2-AS1	AL139147.1	MACC1-AS1	AC083805.2	AC005757.1
AL360093.1	AC025857.2	AL121845.4	AP000593.3	AC040934.1
AC064836.1	MCF2L-AS1	AC090739.1	AC008026.3	AC016949.1
AC005062.1	AC020915.2	AC083906.3	AC009093.1	Z82217.1
PLCG1-AS1	AC245100.7	AC007336.1	AC145285.6	AC012676.1
AC099811.5	AC105339.3	AC116351.2	AP000763.3	AL031770.1
AL354726.1	AL512656.2	AC016831.1	AL020997.2	AC010524.1
AC068790.6	SCAT2	AL121895.1	FAM78B-AS1	CD44-AS1
AL135924.2	AC107081.2	AL133243.3	AC087521.2	AC006017.1
RSF1-IT1	AC025031.4	AC012360.1	GK-AS1	FTX
AC246787.2	AC009996.1	AC005534.1	AC108727.1	AC019118.1
AC138466.1	AC092171.1	AL356124.1	DTNB-AS1	
AL390961.2	AC073487.1	AC021851.1	AC026356.1	
AC073529.1	AC113143.1	AL357054.4	AC127164.1	
PTPRG-AS1	PPP1R14B-AS1	SNHG4	RDH10-AS1	
PEF1-AS1	AL138963.1	AC105339.2	AL031716.1	
LINC02487	AL121832.2	AC020661.3	AC015813.1	
EGOT	Z83843.1	AC008121.3	AL929236.1	
AC104984.3	AC079385.1	INO80-AS1	AC007639.1	
LINC01876	AL080317.1	EPN2-AS1	LINC02604	
AC004837.2	AC009032.1	AC087222.1	LINC00624	
WASHC5-AS1	AC005674.1	AC093627.6	AL133330.1	
AP000695.2	SNHG15	AC073326.1	MIR181A2HG	
AC211476.4	AC068790.5	DLGAP4-AS1	RPS6KA2-IT1	
AC068790.3	CASC15	SCARNA9	AC027279.1	
AC024451.4	LINC01473	ALMS1-IT1	LNCTAM34A	
AC027808.2	AC027288.3	AC026470.2	AC020900.1	
AC015987.1	AC083806.2	AC134312.5	AP000786.1	
AC092681.2	AC104958.2	AC007216.2	AC073592.1	
AC005726.2	AL512306.3	AC022973.5	AC090116.1	
RNF139-AS1	GTF3C2-AS1	AC078860.2	AC007728.3	
AC245128.3	MPRIP-AS1	AC011405.1	MHENCR	
CARNMT1-AS1	AC130371.2	LINC00941	AL355312.3	
Z69666.1	DIAPH2-AS1	N4BP2L2-IT2	AC092436.3	
AC004988.1	LINC01615	AL031005.1	AC131971.1	
AC078852.1	AL359880.1	AC013652.1	ARHGEF38-IT1	
AL807757.2	AC055811.1	C2orf27A	AL365361.1	
AC112512.1	AC100774.1	GLYCTK-AS1	AL133243.2	
Z68871.1	AL136115.2	MAP3K5-AS1	C5orf66-AS1	
MNX1-AS1	AC092168.2	MYOSLID	LINC02595	
AC099811.1	AC007497.1	CDC42-IT1	AC079684.2	
AP001429.1	AL450344.3	AC068790.4	ST7-OT4	
AC244093.5	SLC7A11-AS1	AL596247.1	AC016597.2	
MIR3945HG	AC020663.3	AC026356.2	TSPOAP1-AS1	
AC108463.2	AC069222.1	AL008718.3	Z99289.2	
AP002907.1	AL132989.2	ERCC8-AS1	ZNF451-AS1	
AC004943.3	AC108463.3	AL365436.2	AC006111.2	
AL021578.1	TUSC8	AL121782.1	AC138393.3	
AC090197.1	AC026368.1	AC020612.3	AC084117.1	
AC004594.1	AC009269.5	AL031710.2	AC078883.2	

AC009404.1	AC010655.2	AL137244.1	AP001527.2
AC110769.2	AC012085.2	NEAT1	AP003086.1
SLC16A1-AS1	AL442128.2	LINC01572	AC078795.1
AC132192.1	AC131934.1	EIF1AX-AS1	AC018926.2
AL442067.2	AL157938.3	AC104365.2	LINC01315
FARSA-AS1	FSIP2-AS2	AC100821.2	AC010976.1
ADAMTS9-AS1	LURAP1L-AS1	AL360091.1	ITPK1-AS1
AL357874.2	MAL2-AS1	MMP2-AS1	AP000866.2
AL365356.1	AC103740.1	AP005899.1	SLC12A5-AS1
AC108058.1	GSTCD-AS1	AC018682.1	ITPRIP-AS1
AL117379.1	CSNK1G2-AS1	AL354993.1	SNHG17
AL391421.1	AC009704.2	ARNTL2-AS1	EXTL3-AS1
AC007314.1	AL353804.2	AC124283.3	AC007938.3
AP002812.3	AL442067.1	VPS9D1-AS1	AL031673.1
BX284668.6	KLF7-IT1	LINC00894	AC010320.4
AF117829.1	EIPR1-IT1	AL513327.2	AC012213.3
AL031667.3	AL162724.2	AC055717.2	SLC12A9-AS1
MIR17HG	AL078587.1	KCNQ1OT1	AC112496.1
AL445222.2	AC010973.2	AC130650.2	AC009299.2
AC245884.10	AC022165.1	AC004943.1	AL590723.1
AC005072.1	AL110115.1	AC004691.1	KANSL1L-AS1
AC016027.1	AC008456.1	AC093788.1	LINC02345
FSIP2-AS1	SND1-IT1	AC048344.4	AC007285.2
AC022079.1	BCL2L1-AS1	AC004223.2	AC134407.1
AC109992.2	AC006960.4	DLGAP1-AS5	AC016831.4
AL442125.2	AC113361.1	AF230666.2	AC145423.3
AC036108.3	AL355802.3	AC104695.4	AC007014.2
ALG13-AS1	MBNL1-AS1	AL161729.4	AC004832.4
ZFAS1	AL109614.1	AC004466.2	AL359922.2
Z98885.3	BX640514.2	MRTFA-AS1	AC010168.1
DLEU2	AC007991.4	LINC01091	AL133230.2
LINC-PINT	PABPC4-AS1	AL035071.2	AC012020.1
AC211433.1	CFAP44-AS1	HMGA2-AS1	AL136115.1
AC092127.1	AL158071.1	AC004832.5	AC000061.1
AL031666.2	AC093752.2	LINC02195	AC105429.1
AC005096.1	AL049539.1	AC107993.1	MALAT1
ASAP1-IT2	AC245884.8	TMEM147-AS1	AC006460.1
Z82243.1	AC004466.3	AL021328.1	MRAP-AS1
AC092338.1	AC016394.3	AC026401.2	GAS8-AS1
AL161891.1	MYB-AS1	AC002306.1	ATP2A1-AS1
TFAP2A-AS1	RNASEH2B-AS1	AF124730.2	ACSL3-AS1
AC007684.2	AC078778.1	AC108063.1	AP000866.6
LINC02156	AC078962.2	AC131953.2	FOCAD-AS1
AC004241.4	AC020978.3	AC005479.2	AC073333.1
AL033504.1	ODF2-AS1	TTC3-AS1	AC244093.4

Table S4. The results of gene-set enrichment analysis (GSEA).

Description	setSize	enrichmentScore	NES	pvalue	p.adjust
EPITHELIAL_MESENCHYMAL_TRANSITION	200	0.82578427	3.053513445	1.00E-10	1.77E-09
ECM_RECEPTOR_INTERACTION	84	0.78175221	2.585361842	1.00E-10	1.77E-09
FOCAL_ADHESION	199	0.66787056	2.474296794	1.00E-10	1.77E-09
SYSTEMIC_LUPUS_ERYTHEMATOSUS	55	0.76259041	2.358483063	2.18E-10	3.58E-09
TNFA_SIGNALING_VIA_NFKB	199	0.62075313	2.299738247	1.00E-10	1.77E-09
ANGIOGENESIS	36	0.78073626	2.210791835	1.00E-07	8.86E-07
MYOGENESIS	199	0.58756706	2.176791999	1.00E-10	1.77E-09
INFLAMMATORY_RESPONSE	200	0.58329712	2.156865475	1.00E-10	1.77E-09
COMPLEMENT_AND_COAGULATION_CASCADES	69	0.67966626	2.142160128	1.99E-08	2.29E-07
APICAL_JUNCTION	200	0.56982416	2.107046366	1.00E-10	1.77E-09
ASTHMA	28	0.76537452	2.060971019	7.95E-06	4.69E-05
COAGULATION	138	0.58209347	2.053402845	1.24E-08	1.50E-07
AUTOIMMUNE_THYROID_DISEASE	50	0.66852151	2.035252347	7.65E-06	4.63E-05
LEISHMANIA_INFECTION	69	0.64268082	2.025590086	1.02E-06	8.40E-06
CYTOKINE_CYTOKINE_RECEPTOR_INTERACTION	262	0.52882653	2.014499514	1.00E-10	1.77E-09
CELL_ADHESION_MOLECULES_CAMS	131	0.57675196	2.012832686	3.47E-08	3.80E-07
GRAFT_VERSUS_HOST_DISEASE	37	0.70655079	2.0007368	1.96E-05	0.000107
VIRAL_MYOCARDITIS	68	0.63488297	2.000250717	4.79E-06	3.06E-05
ALLOGRAFT_REJECTION	206	0.53389095	1.98883939	2.25E-09	3.23E-08
HEMATOPOIETIC_CELL_LINEAGE	85	0.60032854	1.984465795	1.83E-06	1.31E-05
TYPE_I_DIABETES_MELLITUS	41	0.65862499	1.93409759	0.000188	0.000771
KRAS_SIGNALING_UP	199	0.51834241	1.920331616	4.52E-08	4.73E-07
COMPLEMENT	199	0.51525611	1.908897653	7.54E-08	7.22E-07
TOLL_LIKE_RECEPTOR_SIGNALING_PATHWAY	102	0.56549834	1.908722036	2.83E-06	1.86E-05
GLYCOSAMINOGLYCAN_BIOSYNTHESIS_CHONDROITIN_SULFATE	22	0.75216112	1.907406748	0.000143	0.000622
UV_RESPONSE_DN	143	0.52702913	1.868255047	1.07E-06	8.48E-06
HYPERTROPHIC_CARDIOMYOPATHY_HCM	83	0.5556309	1.83275021	6.12E-05	0.000287
DILATED_CARDIOMYOPATHY	90	0.55039254	1.829325172	3.69E-05	0.000185
HYPOXIA	200	0.48680492	1.800065004	1.82E-06	1.31E-05
ANTIGEN_PROCESSING_AND_PRESENTATION	80	0.55140952	1.797471375	0.000105	0.000463
IL6_JAK_STAT3_SIGNALING	86	0.53090494	1.759111163	0.000243	0.000981
HEDGEHOG_SIGNALING	36	0.61894142	1.752641352	0.002202	0.006492
PRION_DISEASES	35	0.62291898	1.746298699	0.00253	0.007364
SMALL_CELL_LUNG_CANCER	84	0.52320579	1.730313346	0.00028	0.001111
INTERFERON_GAMMA_RESPONSE	197	0.4634128	1.714307016	2.22E-05	0.000119
LEUKOCYTE_TRANSENDOTHELIAL_MIGRATION	116	0.48734154	1.684266703	0.000449	0.001695
REGULATION_OF_ACTIN_CYTOSKELETON	212	0.43688476	1.630280623	5.00E-05	0.000239
ARRHYTHMOGENIC_RIGHT_VENTRICULAR_CARDIOMYOPATHY_ARVC	74	0.50462095	1.614651184	0.002982	0.008467
IL2_STAT5_SIGNALING	199	0.43391989	1.607566868	0.000155	0.000658
INTESTINAL_IMMUNE_NETWORK_FOR_IGA_PRODUCTION	45	0.53872678	1.597328663	0.010409	0.026309
JAK_STAT_SIGNALING_PATHWAY	155	0.44826447	1.595475045	0.000502	0.001765
PATHWAYS_IN_CANCER	325	0.40672852	1.585988802	3.39E-05	0.000173
VASCULAR_SMOOTH_MUSCLE_CONTRACTION	115	0.45882001	1.58317878	0.001894	0.005731
APICAL_SURFACE	44	0.5308072	1.578930171	0.014153	0.034266
CHEMOKINE_SIGNALING_PATHWAY	186	0.42946898	1.578199103	0.000314	0.001226
MAPK_SIGNALING_PATHWAY	267	0.39006559	1.487196361	0.000597	0.00205
AXON_GUIDANCE	129	0.41948036	1.464440703	0.005795	0.015321
NATURAL_KILLER_CELL_MEDIATED_CYTOTOXICITY	131	0.41752492	1.457139042	0.007026	0.017956
PANCREATIC_CANCER	70	0.45822062	1.454232261	0.016671	0.039933
MITOTIC_SPINDLE	197	0.39194033	1.449908273	0.004534	0.012269
APOPTOSIS	226	0.3575244	1.33937443	0.011823	0.02924

NEUROACTIVE_LIGAND_RECEPTOR_INTERACTION	271	0.33970922	1.298192226	0.017014	0.039933
DNA_REPAIR	149	-0.37021259	-1.349999599	0.021441	0.047877
OOCYTE_MEIOSIS	112	-0.40969165	-1.422214174	0.017377	0.040371
CHOLESTEROL_HOMEOSTASIS	73	-0.44969737	-1.460997352	0.017885	0.040727
GLYCEROPHOSPHOLIPID_METABOLISM	77	-0.44561726	-1.466000972	0.019751	0.044536
ADIPOGENESIS	198	-0.39720629	-1.486620145	0.001261	0.004028
E2F_TARGETS	198	-0.40405873	-1.512266733	0.000822	0.002739
PROTEIN_SECRETION	96	-0.45262224	-1.545870988	0.004368	0.011961
ESTROGEN_RESPONSE_LATE	200	-0.4142171	-1.553543008	0.00046	0.001707
PENTOSE_PHOSPHATE_PATHWAY	26	-0.60054299	-1.585402825	0.017833	0.040727
OTHER_GLYCAN_DEGRADATION	16	-0.66990324	-1.585709553	0.017015	0.039933
PROTEASOME	44	-0.53740785	-1.596831479	0.00693	0.01791
MATURITY_ONSET_DIABETES_OF_THE_YOUNG	25	-0.60896809	-1.600764845	0.010578	0.026444
TRYPTOPHAN_METABOLISM	39	-0.54653256	-1.608927986	0.012942	0.031667
PPAR_SIGNALING_PATHWAY	69	-0.49730294	-1.612201153	0.004797	0.012828
HISTIDINE_METABOLISM	29	-0.61281309	-1.676686568	0.006613	0.017285
ARACHIDONIC_ACID_METABOLISM	57	-0.54043507	-1.679821204	0.002839	0.008163
ARGININE_AND_PROLINE_METABOLISM	54	-0.54832307	-1.681612872	0.001666	0.005109
N_GLYCAN_BIOSYNTHESIS	46	-0.56849881	-1.709797712	0.003455	0.009574
GLYCOLYSIS_GLUCONEOGENESIS	62	-0.5427393	-1.71061954	0.001144	0.003705
SELENOAMINO_ACID_METABOLISM	25	-0.65811751	-1.729961534	0.003317	0.009303
GLUTATHIONE_METABOLISM	47	-0.58388467	-1.757463281	0.000975	0.003204
STEROID_HORMONE_BIOSYNTHESIS	55	-0.57505607	-1.773196466	0.000495	0.001765
OLFACTORY_TRANSDUCTION	377	-0.44611547	-1.7763898	1.12E-08	1.44E-07
GLYCOSYLPHOSPHATIDYLINOSITOL_GPI_ANCHOR_BIOSYNTHESIS	25	-0.67782932	-1.78177703	0.001612	0.005009
XENOBIOTIC_METABOLISM	197	-0.48069417	-1.797261444	1.27E-06	9.72E-06
NITROGEN_METABOLISM	23	-0.69568564	-1.808619561	0.002109	0.006298
LINOLEIC_ACID_METABOLISM	29	-0.66262572	-1.812976354	0.001319	0.004156
BILE_ACID_METABOLISM	112	-0.52963631	-1.838593169	3.31E-05	0.000173
PYRUVATE_METABOLISM	40	-0.62924623	-1.845007347	0.000506	0.001765
PORPHYRIN_AND_CHLOROPHYLL_METABOLISM	40	-0.63439272	-1.860097323	0.000399	0.00153
ALZHEIMERS_DISEASE	163	-0.51055431	-1.874470864	8.13E-07	6.92E-06
PROXIMAL_TUBULE_BICARBONATE_RECLAMATION	23	-0.7223121	-1.877842113	0.000776	0.002624
STEROID_BIOSYNTHESIS	17	-0.7914498	-1.908148748	0.000487	0.001765
STARCH_AND_SUCROSE_METABOLISM	51	-0.62745709	-1.91279136	9.63E-05	0.000434
HUNTINGTONS_DISEASE	177	-0.51900654	-1.920515372	9.14E-08	8.41E-07
DRUG_METABOLISM_OTHER_ENZYMES	51	-0.64057017	-1.952766336	4.79E-05	0.000235
TERPENOID_BACKBONE_BIOSYNTHESIS	15	-0.82975216	-1.963354511	0.000166	0.000695
PROPANOATE_METABOLISM	32	-0.71127268	-1.98669189	6.41E-05	0.000295
VALINE_LEUCINE_AND_ISOLEUCINE_DEGRADATION	44	-0.68564301	-2.037291322	7.60E-06	4.63E-05
PENTOSE_AND_GLUCURONATE_INTERCONVERSIONS	28	-0.76932668	-2.087341851	1.34E-05	7.50E-05
CITRATE_CYCLE_TCA_CYCLE	30	-0.76612792	-2.108093526	1.03E-05	5.94E-05
MYC_TARGETS_V1	195	-0.56544548	-2.117494945	1.00E-10	1.77E-09
ASCORBATE_AND_ALDARATE_METABOLISM	25	-0.81008478	-2.129430533	2.11E-06	1.47E-05
BUTANOATE_METABOLISM	34	-0.76089247	-2.161449923	2.81E-06	1.86E-05
PARKINSONS_DISEASE	125	-0.61864314	-2.186475009	2.09E-09	3.21E-08
FATTY_ACID_METABOLISM	172	-0.59586873	-2.198973786	1.00E-10	1.77E-09
METABOLISM_OF_XENOBIOTICS_BY_CYTOCHROME_P450	68	-0.68150433	-2.205324138	5.12E-08	5.12E-07
DRUG_METABOLISM_CYTOCHROME_P450	70	-0.69807096	-2.251792904	3.06E-09	4.14E-08
PEROXISOME	144	-0.62708336	-2.272124449	1.00E-10	1.77E-09
RETINOL_METABOLISM	64	-0.75212148	-2.401058389	1.00E-10	1.77E-09
OXIDATIVE_PHOSPHORYLATION	246	-0.65533659	-2.517434499	1.00E-10	1.77E-09
